# Low apolipoprotein A-II levels causally contribute to increased mortality in septic shock

**DOI:** 10.1186/s40560-025-00782-2

**Published:** 2025-02-20

**Authors:** Nozomi Takahashi, Kyle R. Campbell, Tadanaga Shimada, Taka-aki Nakada, James A. Russell, Keith R. Walley

**Affiliations:** 1 Centre for Heart Lung Innovation, St. Paul’s Hospital, The University of British Columbia, 1081 Burrard Street, Vancouver, BC V6Z 1Y6 Canada; 2 Department of Emergency and Critical Care Medicine, Chiba University Graduate School of Medicine, Chiba, Japan

**Keywords:** Sepsis, Septic shock, Lipids, Apolipoprotein, Cholesterol

## Abstract

**Background:**

Lipoproteins and their component apolipoproteins play an important role in sepsis. However, little is known with regard to the association and causal contribution of these proteins to mortality in patients of different ancestries following septic shock. The objective of this study was to determine whether lipoprotein and apolipoprotein levels, and related genetic variants, are associated with clinical outcomes in septic shock.

**Methods:**

We investigated the association between lipoprotein and apolipoprotein levels at the point of admission to the intensive care unit and in-hospital mortality in 687 Japan patients diagnosed with septic shock. For each clinically significant candidate protein, we extracted haplotype tag single nucleotide polymorphisms (SNPs) of the corresponding gene and examined the association of the candidate gene variants with 28-day mortality and organ dysfunction. We tested for replication in a Caucasian septic shock cohort (Vasopressin and Septic Shock Trial, VASST, n = 474). To determine whether the candidate lipoprotein causally contributed to septic shock outcome, we used a Mendelian randomization analysis based on polygenic scores generated from a genome-wide association study (GWAS) in the Japan cohort.

**Results:**

In the Japan cohort, low apolipoprotein A-II levels were associated with increased septic shock mortality (adjusted odds ratio, 1.05; 95%CI, 1.02–1.09; *P* < 0.001). For a haplotype tag SNP of the corresponding *ApoA2* gene, rs6413453 GG carriers had significantly higher 28-day mortality (adjusted hazard ratio [aHR], 1.79; 95% confidence interval [CI], 1.06–3.04; *P* = 0.029) and significantly fewer days free of cardiovascular, respiratory, renal and neurologic dysfunction than AG/AA carriers. This result was replicated in the Caucasian septic shock cohort (28-day mortality: aHR, 1.65; 95% CI, 1.02–2.68; *P* = 0.041). Mendelian randomization using 9 SNPs from an apolipoprotein A-II GWAS suggested that genetically decreased levels of apolipoprotein A-II were a causal factor for increased mortality in septic shock (odds ratio for mortality due to a 1 mg/dL decrease in apolipoprotein A-II is 1.05 [95% CI; 1.01–1.03, *P* = 0.0022]).

**Conclusions:**

In septic shock, apolipoprotein A-II levels and *ApoA2* genetic variations are important factors associated with outcome.

**Supplementary Information:**

The online version contains supplementary material available at 10.1186/s40560-025-00782-2.

## Background

Despite recent advances in understanding, sepsis remains a major cause of mortality representing 20% of all global deaths and accounting for over 40% of all deaths in intensive care units (ICUs) [1–3]. Lipopolysaccharide (LPS), lipoteichoic acid (LTA), and other pathogen lipid toxins activate the septic inflammatory response by binding to innate immune receptors. LPS and LTA are carried within cholesterol particles including high-density lipoprotein cholesterol (HDL), low-density lipoprotein cholesterol (LDL), and very low-density lipoprotein cholesterol (VLDL) [4]. HDL has a particularly high affinity for binding and sequestration of LPS and LTA, thereby mitigating their excessive innate immune activation in sepsis [5, 6]. Apolipoprotein A-I and A-II are major components of HDL and play a role in pathogen lipid binding. These lipoproteins also interact with lipid rafts on cell membranes and are often present on immune cell receptors to modulate immune responses to pathogen lipids [7, 8].

Recently, a number of investigations have sought to elucidate the pathophysiology of lipoproteins in sepsis and translate this into novel treatment [9, 10]. Study of genetic variants in cholesterol pathway genes has provided insight into the effects of lipoproteins during sepsis and a number of studies have highlighted the central and causal role of HDL in mitigating the septic inflammatory response [11–13]. However, few studies have focused on the apolipoproteins within HDL, in part, because apolipoproteins are rarely measured in sepsis, despite being important pathogen lipid-binding components of HDL.

We hypothesized that HDL and its constituent apolipoproteins would be associated with mortality in septic shock. We sought to examine their contribution to mortality and organ dysfunction in two ancestrally diverse septic shock cohorts [14]. To estimate the causal effects on outcome, we performed Mendelian randomization using candidate lipoproteins or apolipoproteins as exposures.

## Materials and methods

### Study design and definition

This study was a retrospective observational study. Septic shock was defined according to Sepsis-3 as infection causing a Sequential Organ Failure Assessment (SOFA) score of 2 or more and the need for a vasopressor to maintain necessary blood pressure after adequate volume resuscitation [1]. First, we analyzed the relationship between lipoproteins and apolipoproteins on in-hospital mortality using data from a Japan cohort of septic shock patients who had measurements of lipoprotein and apolipoprotein concentrations at ICU admission. Through this analysis, we detected candidates that were significantly associated with in-hospital mortality. Subsequently, we identified haplotype tag single nucleotide polymorphisms (SNPs) of the genes corresponding to the candidate proteins and examined whether these SNPs were significantly associated with the 28-day mortality and organ dysfunction in two septic shock cohorts of different ancestry. Through the use of a Mendelian randomization strategy, we elucidated whether the candidate lipoprotein or apolipoprotein could be a causal factor in determining septic shock outcome. Procedures were followed in accordance with the ethical standards of the responsible committee on human experimentation (institutional or regional) and with the Helsinki Declaration of 1975.

### Derivation (Japan) cohort

We screened a total of 899 sepsis patients who were admitted to ICU at Chiba University Hospital, Chiba, Japan, between October 2012 and January 2022. Of these, 687 patients were diagnosed with septic shock and had their lipoprotein and apolipoprotein levels measured. Of these, 513 patients further have consented to genetic analysis. All of the patients who provided specimens had Japan ancestry.

This study was approved by the Institutional Review Board of Chiba University Graduate School of Medicine and was performed in accordance with the committee’s guidelines including genetic analysis; written informed consent was obtained from all patients or their authorized representatives (approval number, 959 [approval date, 9 June 2022; study title, “Genetic polymorphisms affecting the clinical course of sepsis”]).

### Validation (VASST) cohort

VASST was a multicenter (27 ICUs in Canada, Australia, and the United States), randomized, double-blind trial conducted between July 2001 and April 2006 in a total of 778 septic shock patients [15]. This work was completed prior to Sepsis-3 being defined. However, patients with septic shock in this study had a proven or suspected infection, developed new organ dysfunction, had hypotension, and required vasopressors despite adequate fluid resuscitation. Therefore, all patients in this cohort satisfied the Sepsis-3 septic shock definition. To reduce the potential for population stratification and investigate whether genetic variants were associated with clinical outcomes across ancestry, this analysis was restricted to 474 Caucasian ancestry patients who had DNA available. Lipoprotein and apolipoprotein measurements were not available but genome-wide genotyping was available in this cohort and as such, was used for validation of the genetic analysis. The Institutional Review Board of St. Paul’s Hospital and the University of British Columbia approved this study, which was performed in accordance with the committee’s guidelines (approval number, H23-03069 [approval date, 7 February 2024; study title, “DSIRS Registry”] and H02-50076 [approval date, 9 July 2002; study title, “Translational research to improve sepsis outcomes”]). Written informed consent was obtained from all patients or their authorized representatives.

Selection of SNPs and genotyping are described in a Supplementary file. Haplotype tag SNPs for the candidate gene were identified from the gene region extending from 2,000 bp upstream of the 5’ untranslated region (UTR) to 2000 bp downstream of the 3’ UTR.

### Statistical analyses

The primary outcome was 28-day mortality. Univariate analyses were first performed for lipoprotein/apolipoprotein between survivors and non-survivors. The results were checked for robustness by Bonferroni correction, taking into account the effect of multiple testing due to repeated testing. Furthermore, candidate lipoprotein/apolipoprotein associations were examined by multivariate analysis adjusted for age, gender, APACHE II score and SOFA score, considering the influence of confounding factors. For candidates with significant results, a genetic analysis was performed as a next step. RegulomeDB, a database for prioritizing functionally important single nucleotide polymorphisms (SNPs) located in non-coding regions of the human genome, was used to assess the candidate SNPs. This provides information on regulatory elements such as gene expression and transcription factor (TF) binding sites. For the analysis, a Cox proportional hazard model was used to test for a difference in hazard of death over 28 days by genotype corrected for potential confounding factors including age, gender, and chronic steroid use in both Japan and VASST cohorts. Chronic steroid use was chosen for the adjustment in the analysis as a confounder since it may potentially affect the gene expression of lipoproteins [16]. The secondary outcomes were days alive and free from organ dysfunctions during the first 28 days according to the Brussels criteria (Supplementary file; Table S1) [15, 17]. In the clinical analysis for lipoproteins and apolipoproteins, a logistic regression model was used to adjust for the effect of age, gender, Acute Physiology and Chronic Health Evaluation (APACHE) II score, and SOFA score.

To evaluate the causal effect of candidate lipoproteins or apolipoproteins on outcome a Mendelian randomization analysis was performed (‘MendelianRandomization’ [v0.9.0] and ‘TwoSampleMR’ [v0.5.8] package for R). This analysis used the strongly associated SNPs obtained from the result of a genome-wide association study (GWAS) as statistical instruments [18]. These relevant SNPs were obtained from the Japan cohort by performing a GWAS using candidate protein values as quantitative traits. In the performed GWAS, minor allele frequencies of 5% or less for quantitative traits, *P* values of 10^–6^ or less for Hardy–Weinberg equilibrium tests, and linkage disequilibrium coefficient *r*^2^ values of 0.01 or greater were excluded from the analysis for quality control. Obtained beta coefficients for the candidate protein were calculated using a generalized linear model adjusted for age and gender and created by its beta and its standard error values. In the Mendelian randomization analysis, we used an inverse-variance weighted (IVW) model. Potential bias due to uncorrelated horizontal pleiotropy or heterogeneity was estimated using the MR-Egger intercept test and Cochran’s Q test. Weighted median mode was also used to evaluate correlated horizontal pleiotropy. Meta-analysis to integrate the Mendelian randomization results of the Japan and VASST cohorts was performed using a fixed effects model with inverse variance method using the R “meta” package.

To investigate the impact of baseline apolipoprotein A-II levels on septic shock mortality, we conducted two-sample Mendelian randomization using the protein quantitative trait locus (pQTL) database (Supplementary file; Table S2) [19] and separately, using the whole blood transcriptome database (we accessed GSE95233 [20]). In addition, to examine the effect of *ApoA2* gene expression on outcome, we extracted cis-eQTL variants for *ApoA2* using the eQTL database [21, 22] and used these as genetic variants to analyze the association with 28-day mortality using two-sample Mendelian randomization. This database does not provide beta coefficient and standard error, and requires conversion from Z scores using the European population, so only the VASST cohort was used in the analysis (Supplementary file; Methods, Table S3). The Mendelian randomization analysis performed in the study followed the STROBE-MR statement [23].

Univariate analysis was performed using a Mann–Whitney U test. A two-tailed *P* value of < 0.05 was considered to be statistically significant. Data are presented as medians with interquartile range. All analyses were performed using R version 4.1.2 (R Foundation for Statistical Computing, Vienna, Austria, http://www.R-roject.org/).

## Results

### Clinical features and lipoprotein/apolipoprotein levels

In the Japan septic shock cohort (n = 687) 214 patients died in hospital. Body mass index was significantly lower, and APACHE II and SOFA score was significantly higher in non-survivors (Table 1). In non-survivors, apolipoprotein A-I concentration was lower (survivors 70 mg/dL; non-survivors 60 mg/dL; *P* = 0.007), apolipoprotein A-II was lower (survivors 12.0 mg/dL; non-survivors 9.9 mg/dL; *P* < 0.001), and apolipoprotein C-III was lower (survivors 4.7 mg/dL; non-survivors 4.2 mg/dL; *P* = 0.046). There were no statistically significant differences in HDL (*P* = 0.18) and LDL (*P* = 0.17) but the sum of these components, total cholesterol, was significantly lower in non-survivors (*P* = 0.045). Furthermore, among these lipids, only apolipoprotein A-II remained significant after Bonferroni correction (*P* = 0.006).

**Table 1:** Baseline characteristics in the derivation cohort by hospital mortality (Japan cohort, n = 687)

	Survive (*n* = 473)	Dead (*n* = 214)	*P*-value
Age, yr	69 (57–77)	70 (62–76)	0.18
Male sex, n (%)	307 (64.9)	139 (65.0)	1.00
BMI, kg/m^2^	23 (20–26)	22 (19–25)	0.030
APACHE II score^1^	28 (22–34)	36 (29–40)	< 0.001
SOFA score^2^	12 (9–15)	15 (12–17)	< 0.001
Comorbidity, n (%)			
Diabetes mellitus	122 (25.8)	47 (22.0)	0.29
Dyslipidemia	62 (13.1)	22 (10.3)	0.32
Congestive heart failure	38 (8.0)	25 (11.7)	0.15
End stage renal failure	30 (6.3)	25 (11.7)	0.022
Chronic pulmonary disease	25 (5.3)	18 (8.4)	0.13
Lipid laboratory data on Day1			
HDL cholesterol, mg/dL	29 (18–42)	27 (15–42)	0.18
LDL cholesterol, mg/dL	41 (21–66)	37 (17–62)	0.17
Total cholesterol, mg/dL	105 (79–140)	101 (68–133)	0.045
Apolipoprotein A-I, mg/dL	70 (47–94)	60 (38–84)	0.007
Apolipoprotein A-II, mg/dL	12.0 (8.5–16.8)	9.9 (6.2–13.8)	< 0.001
Apolipoprotein B, mg/dL	54 (38–76)	50 (35–73)	0.18
Apolipoprotein C-III, mg/dL	4.7 (3.1–6.6)	4.2 (2.6–5.6)	0.046
Apolipoprotein E, mg/dL	3.7 (2.9–5.1)	3.8 (2.8–5.0)	0.84

Data are median (interquartile range) for continuous variables

*P* values were calculated using Pearson’s Chi-square test and Mann–Whitney U test

^1^APACHE, Acute Physiology and Chronic Health Evaluation

^2^SOFA, Sequential Organ Failure Assessment

Of these three apolipoproteins, only apolipoprotein A-II showed a significant difference for in-hospital mortality after adjusting for age, gender, APACHE II score and SOFA score (adjusted odds ratio, 0.95; 95% confidence interval [CI], 0.92–0.98; *P* < 0.001) (Supplementary file; Table S4). Therefore, we considered apolipoprotein A-II as a candidate to test for a potential causal contribution to mortality of septic shock using a Mendelian randomization analysis. The next step in this analysis required identification of SNPs to use as genetic instrumental variables, as follows.

### Candidate SNPs of the selected apolipoprotein and altered 28-day mortality

Of the 6 SNPs within 1337 base pairs of the apolipoprotein A-II (*ApoA2*) gene and 2000 base pairs upstream and downstream, five tag SNPs were identified (rs3829793, rs5082, rs5085, rs6413453, rs12721035), and these SNPs contained 99% of the genetic diversity within this gene region and were in Hardy–Weinberg equilibrium, while rs6413453 and rs12721035 is in the linkage disequilibrium in the Japan cohort (Supplementary file; Table S5, Figure S1). In other words, it was determined that almost all SNPs within *ApoA2* are represented by these tag SNPs and independent effects can be investigated. Of the 5 tag SNPs a significant association was found for rs6413453 (Table 2).

**Table 2:** Hazard ratio of 28-day mortality in septic shock cohorts for *ApoA2* variants

	Japan cohort Hazard ratio (95% CI)	*P*-value	VASST cohort Hazard ratio (95% CI)	*P*-value
rs3829793				
Age, per yr	1.02 (1.01–1.04)	0.013	1.02 (1.01–1.04)	0.0014
Male	1.52 (0.89–2.58)	0.12	1.03 (0.74–1.43)	0.87
Chronic steroid use	2.31 (1.28–4.18)	0.0057	1.82 (1.29–2.57)	< 0.001
CC genotype	1.32 (0.80–2.16)	0.27	1.08 (0.78–1.49)	0.66
rs5082				
Age, per yr	1.02 (1.01–1.04)	0.012	1.02 (1.01–1.03)	0.0013
Male	1.51 (0.89–2.57)	0.13	1.03 (0.74–1.43)	0.85
Chronic steroid use	2.33 (1.29–4.21)	0.0053	1.82 (1.29–2.57)	< 0.001
AA genotype	1.17 (0.61–2.22)	0.64	1.60 (0.77–1.48)	0.73
rs5085				
Age, per yr	1.02 (1.01–1.04)	0.012	1.02 (1.01–1.03)	< 0.001
Male	1.51 (0.89–2.57)	0.13	1.04 (0.75–1.45)	0.81
Chronic steroid use	2.32 (1.28–4.19)	0.0055	1.78 (1.26–2.53)	0.0011
CC genotype	0.94 (0.59–1.51)	0.81	0.85 (0.61–1.20)	0.36
rs6413453				
Age, per yr	1.02 (1.00–1.04)	0.015	1.02 (1.01–1.03)	< 0.001
Male	1.57 (0.92–2.66)	0.10	1.03 (0.74–1.43)	0.87
Chronic steroid use	2.22 (1.23–4.03)	0.0083	1.83 (1.30–2.59)	< 0.001
GG genotype	1.79 (1.06–3.04)	0.029	1.65 (1.02–2.68)	0.041
rs12721035				
Age, per yr	1.02 (1.00–1.04)	0.015	1.02 (1.01–1.03)	< 0.001
Male	1.57 (0.92–2.66)	0.10	1.03 (0.74–1.43)	0.87
Chronic steroid use	2.22 (1.23–4.03)	0.0083	1.83 (1.30–2.59)	< 0.001
TT genotype	1.79 (1.06–3.04)	0.029	1.01 (0.57–1.79)	0.98

**Fig. 1:**
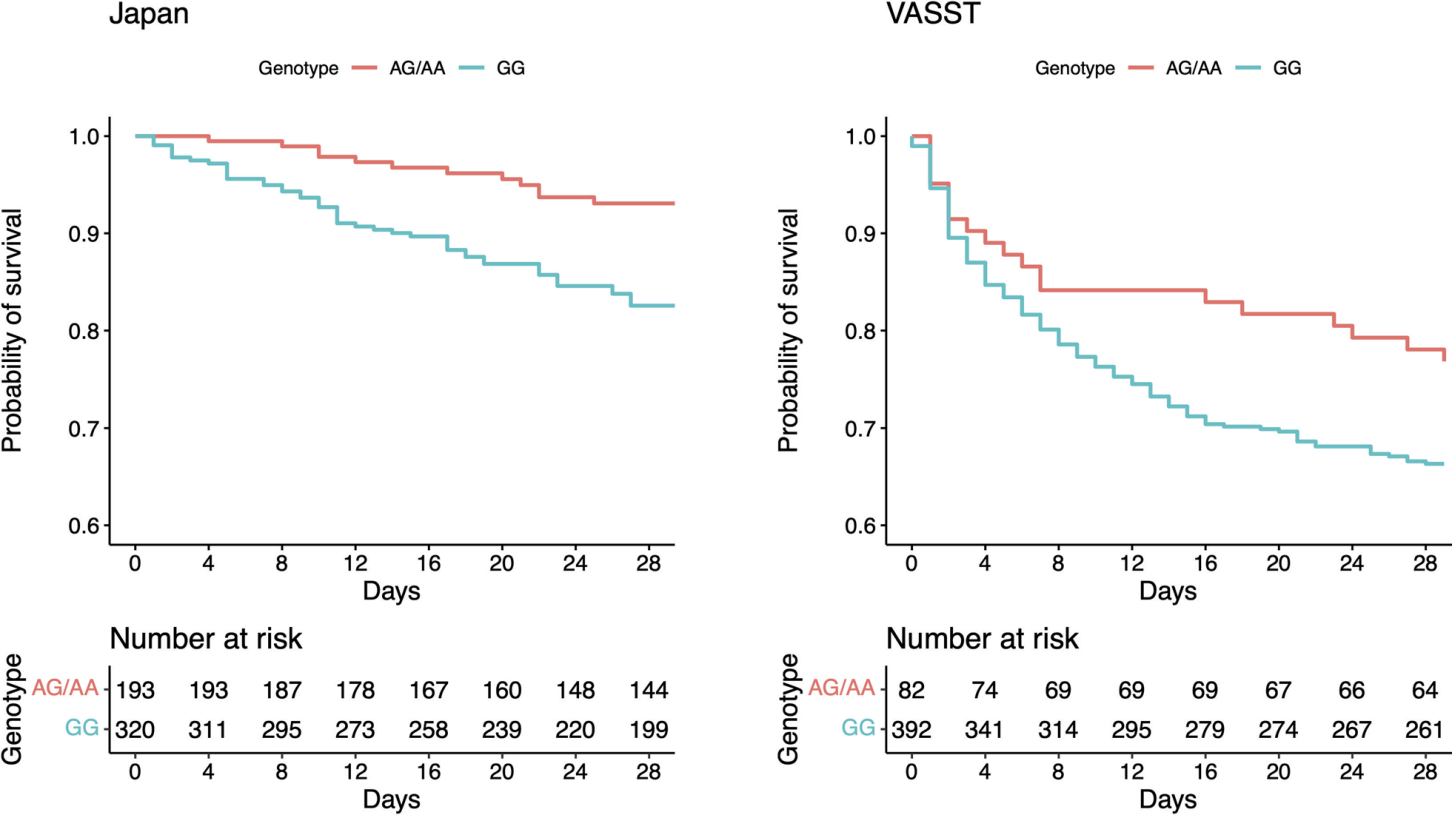
Survival curves for the patients with septic shock in two cohorts by the genotype of *ApoA2* rs6413453 polymorphism. Patients who had rs6413453 GG genotype (dashed line) had significantly decreased survival in the Japan cohort and the VASST cohort. P values were calculated using Cox proportional hazard model corrected for age, gender, and chronic steroid use (Japan: adjusted hazard ratio [HR], 1.79; 95% confidence interval [CI], 1.06–3.04; *P* = 0.029. VASST: adjusted HR: 1.65; 95% CI, 1.02–2.68; *P* = 0.041)

Patients who had *ApoA2* rs6413453 GG genotype had a significant increase in the hazard of death over 28 days in the Japan cohort after adjusted by age, gender and chronic steroid use using Cox proportional hazard model (adjusted hazard ratio [HR], 1.79; 95% CI, 1.06–3.04; *P* = 0.029) (Fig. 1**, **Table 2). This result was replicated in the validation (VASST) cohort (adjusted HR, 1.65; 95% CI, 1.02–2.68; *P* = 0.041), and meta meta-analysis of the Japan and VASST cohorts using random effect model (HR, 1.71; 95% CI, 1.20–2.44; *P* = 0.0031). This result remained significant after Bonferroni correction for the 5 tag SNPs (*P* = 0.016). There was no difference by genotype in baseline characteristics of the patients in the Japan and VASST cohorts except for a lower number of male patients in the Japan cohort (Supplementary file; Table S6).

This SNP, *ApoA2*-rs6413453, is located in the intronic region, and an association with apolipoprotein A-II deficiency was indicated in ClinVar. However, no GWAS with apolipoprotein A-II as a trait existed in the public database, so the effect size of the SNP on measurements of apolipoprotein A-II was unknown, while PhenoScanner suggested a potential influence on the gene expression of *ApoA2*. The ranking score for functionality of “1b” was assigned by RegulomeDB which means this variant has been shown to be associated with eQTLs, TF binding, any motif, DNase Footprint and DNase peak.

### Secondary outcomes by the genotype of *ApoA2* rs6413453 polymorphism

In the Japan cohort, patients with GG genotype had fewer days free of cardiovascular, respiratory, renal and neurologic dysfunction, vasopressor and ventilator support as defined by Brussels criteria compared to AG/GG genotype patients (Table 3). In the VASST cohort, patients with the GG genotype also had fewer days free of cardiovascular, respiratory and renal dysfunction, as well as more vasopressor use, ventilator use, and renal replacement therapy. In the Japan cohort, median apolipoprotein A-II levels were 12.4 (IQR 8.7, 18.7) mg/dL in AG/AA patients and 11.3 (7.7, 15.8) mg/dL in GG genotype patients (*P* = 0.069) (Supplementary file; Figure S3).

**Table 3:** Days alive and free of organ dysfunction and artificial support in septic shock cohorts with the genotype of *ApoA2* rs6413453 polymorphism

*ApoA2* rs6413453 Genotype	Japan cohort (*n* = 513)			VASST cohort (*n* = 474)		
	GG (*n* = 320)	AG/AA (*n* = 193)	*P*-value	GG (*n* = 392)	AG/AA (*n* = 82)	*P*-value
Organ dysfunction^1)^						
Cardiovascular	24 (13–27)	26 (22–28)	< 0.001	19 (0–24)	22 (12–24)	0.009
Respiratory	10 (0–21)	16 (2–23)	< 0.001	3 (0–15)	8 (0–18)	0.020
Renal	23 (3–28)	27 (19–28)	< 0.001	21 (3–28)	26 (10–28)	0.008
Coagulation	22 (5–26)	23 (15–27)	0.12	25 (7–28)	28 (14–28)	0.16
Hepatic	26 (6–28)	27 (15–28)	0.31	27 (7–28)	28 (13–28)	0.085
Neurologic	20 (4–26)	22 (14–27)	0.020	16 (0–24)	19 (6–25)	0.31
Artificial support						
Vasopressor	22 (8–25)	24 (16–27)	0.001	19 (0–24)	23 (12–25)	0.008
Ventilator	17 (2–24)	20 (11–24)	0.020	8 (0–20)	14 (2–21)	0.045
Renal replacement therapy	23 (7–28)	25 (19–28)	0.064	25 (6–28)	28 (15–28)	0.008
Total free days	1 (0–18)	4 (0–17)	0.21	0 (0–8)	4 (0–13)	0.007

Data are median (interquartile range) for continuous variables. *P* values were calculated using Mann–Whitney U test

^1^Organ dysfunction was recorded if the patient met the Brussels organ dysfunction criteria (moderate, severe, or extreme)

### Causal inference of apolipoprotein A-II on septic shock survival

Mendelian randomization was used to test for causal association between low apolipoprotein A-II levels on septic shock and increased 28-day mortality. Since there were no GWAS results available for apolipoprotein A-II on septic shock in previous databases, we performed an age- and sex-adjusted GWAS using the Japan cohort. Using the conventional threshold *P* < 10^–5^ to identify suggestive SNP associations, a total of 9 SNPs were identified as instruments (Supplementary file; Table S7).

**Fig. 2:**
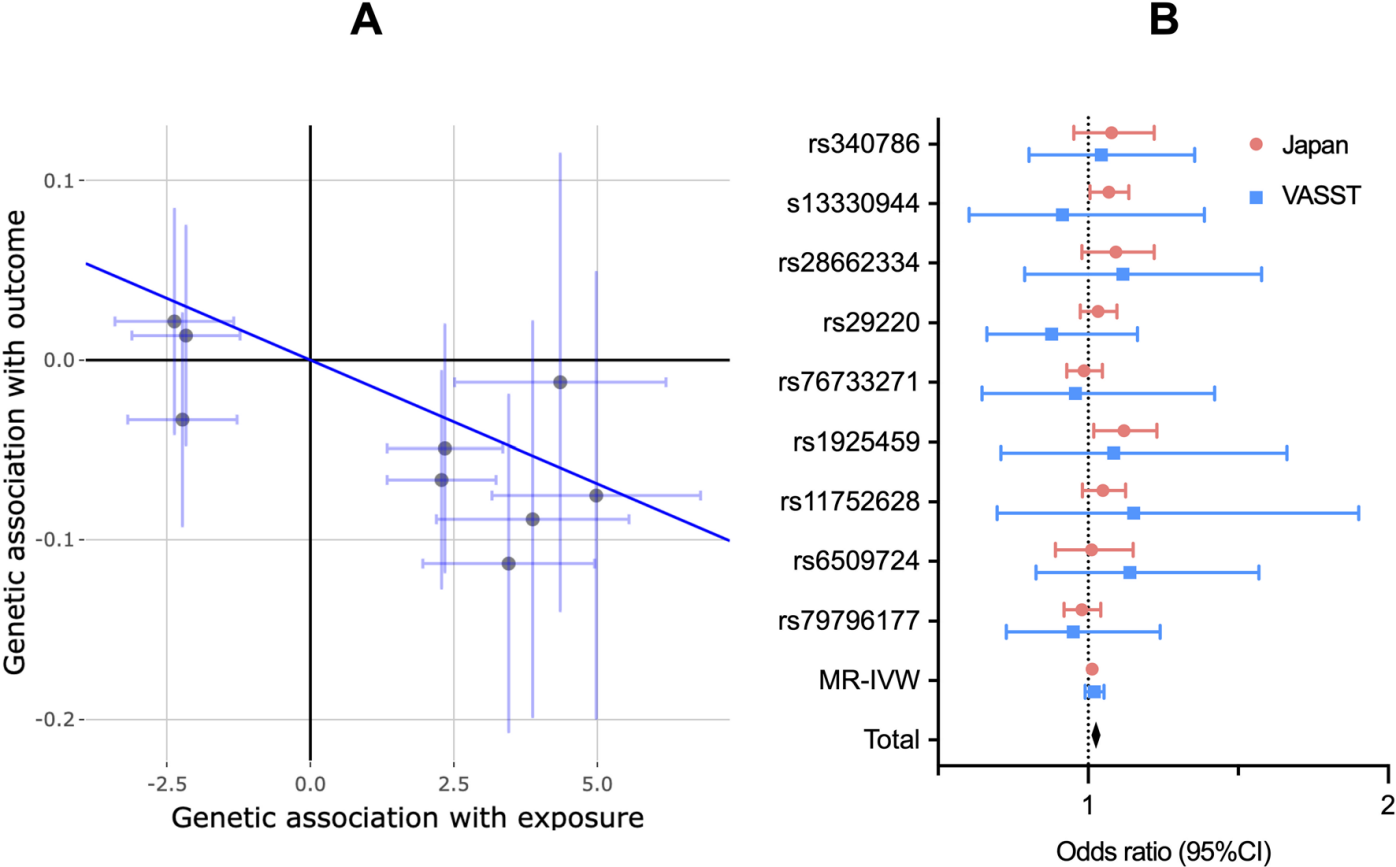
Mendelian randomization analyses for the change in 28-day mortality in septic shock. **A** Inverse variance weighted model. The odds ratio of 28-day mortality to a decrease in apolipoprotein A-II 1 mg/dL was 1.03 (95% CI; 1.01–1.05, *P* = 0.009). **B** A two-sample Mendelian randomization to the VASST cohort using effect sizes for apolipoprotein A-II from the Japan cohort. The odds ratio of 28-day mortality for a decrease in apolipoprotein A-II concentration of 1 mg/dL was 1.03 (95% CI; 1.00–1.04, *P* = 0.21). In a meta-analysis, the integrated odds ratio was 1.02 (95% CI; 1.01–1.03, *P* = 0.0022)

In the Mendelian randomization analysis, the odds ratio of 28-day mortality for a decrease in apolipoprotein A-II concentration of 1 mg/dL was 1.02 (95% CI; 1.01–1.03, *P* = 0.006) and Cochran’s Q statistic was 9.2 for the Japan cohort, indicating that SNPs associated with increased apolipoprotein A-II significantly reduced 28-day mortality (Fig. 2A). The estimate of the intercept for the MR-Egger method was 0.036 (95% CI, -0.065–0.136; *P* = 0.49) and the odds ratio by weighted median method was 1.02 (95% CI; 1.01–1.03, *P* = 0.025), which suggested that the results were not influenced by either uncorrelated and correlated horizontal pleiotropy (Supplementary file; Figure S4). These results must be taken with caution since both genetic instruments, exposure (apolipoprotein A-II levels) and outcome (mortality) were all conditioned on recruitment as a selective bias, and the possibility of violating Mendelian randomization’s assumptions could not be completely ruled out by these tests. A two-sample Mendelian randomization to the VASST cohort using effect sizes for apolipoprotein A-II from the Japan cohort showed that the odds ratio of 28-day mortality for a decrease in apolipoprotein A-II concentration of 1 mg/dL was 1.03 (95% CI; 1.00–1.04, *P* = 0.21). In a meta-analysis, the integrated odds ratio was 1.02 (95% CI; 1.01–1.03, *P* = 0.0022) (Fig. 2B).

A two-sample Mendelian randomization using pQTL database showed the same directionality that decreased apolipoprotein A-II related to higher 28-day mortality in both cohorts, but it was not significant (Japan cohort; *P* = 0.72, VASST cohort; *P* = 0.49) (Supplementary file; Figure S5). Furthermore, the results from whole blood transcriptome database showed that *ApoA2* gene expression was significantly upregulated in septic shock in both two probes for *ApoA2* gene expression (mean log2 fold change; 0.97), suggesting the involvement and role of apolipoprotein A-II in septic shock may have a different mechanism during baseline and septic shock (Supplementary file; Table S8).

To address this issue using independent data, we performed a two-sample Mendelian randomized analysis using cis-eQTLs for *ApoA2* expression as genetic variants. Decreased *ApoA2* expression was significantly associated with increased 28-day mortality in IVW (*P* = 0.013) and weighted median mode (*P* = 0.034) (Supplementary file; Figure S6). The results were not significant by MR-Egger (*P* = 0.70) or weighted mode (*P* = 0.79), suggesting that the contribution of *ApoA2* to mortality in septic shock may indicate the importance of protein levels in the disease state contributing to pleiotropy.

## Discussion

In this study, apolipoprotein A-II was found to be significantly lower in septic shock non-survivors even after correction for potential confounders using multivariate analysis. Patients with GG genotype of a tag SNP within the corresponding *ApoA2* gene, rs6413453 G/A, had significantly higher 28-day mortality in two septic shock cohorts of different ancestry. Furthermore, this GG genotype was found to be significantly associated with fewer days free of cardiovascular and pulmonary organ dysfunction. Mendelian randomization suggested that genetically determined low apolipoprotein A-II levels causally contributed to increased 28-day mortality in septic shock. Importantly, these results were replicated in a second septic shock cohort of different ancestry.

A number of studies have demonstrated an association between the concentration of apolipoprotein A-I, which is the major component of HDL, and clinical outcomes of septic shock [24–27]. However, there have been few studies of septic shock focusing on apolipoprotein A-II. The current study is the first to demonstrate an association of apolipoproteins other than apolipoprotein A-I with sepsis and septic shock.

In our study, a decrease in apolipoprotein A-II was associated with adverse outcomes. Apolipoprotein A-II accounts for 20% of HDL protein content and has an important impact on the structural stability of HDL particles and the determination of the correct and functional size of the HDL complex, including apolipoprotein A-I conformation. This is due to apolipoprotein A-II having a higher affinity for lipids as compared with apolipoprotein A-I [27–29]. Apolipoprotein A-II may have altered the stabilization of HDL structure, resulting in increased mortality [10, 30]. Furthermore, apolipoprotein A-II downregulates neutrophil function, suppresses cytokine production, and exerts anti-inflammatory effects [31, 32]. Apolipoprotein A-II inhibits the formation of LPS aggregates which may contribute to controlling the response to LPS [33, 34].

Mendelian randomization using genetic instruments derived from GWAS for apolipoprotein A-II traits in septic shock and, separately, in non-septic subjects, yielded different results suggesting differences in regulation in these distinct states. In sepsis, baseline or genetically predicted baseline HDL levels might not predict prognosis, consistent with our results [35, 36]. In contrast, when *ApoA2* cis-eQTLs were analyzed as genetic variants increased expression was significantly associated with decreased mortality in a Mendelian randomization analysis. Functional changes in HDL may account for these observations [37, 38]. Our study is focused on septic shock, and the results of the QTL analysis emphasize the importance of studying the mechanisms involved in the effects of apolipoprotein A-II in septic shock and its relationship with HDL.

A tag SNP of *ApoA2* gene, rs6413453 G/A, showed an association with altered mortality and organ dysfunctions in both the Japanese derivation and Caucasian validation cohorts. The *ApoA2* gene is located on chromosome 1 in region 1q21-q23 and rs6413453 is in an intronic region of the gene [39]. This SNP has been reported to be a susceptibility locus for type 2 diabetes, while no studies have shown an association with inflammation such as sepsis [40–42]. RegulomeDB scoring of SNP functionality provided a rank of 1b for rs6413453. This indicates that this variant is likely to affect binding and linked to expression of *ApoA2* including alteration of transcription factor binding and a gene regulatory effect. Furthermore, PhenoScaner found a relation to *ApoA2* gene expression in the study of context-dependent eQTLs in whole blood (*P* = 8.78*10^–6^) [43]. Therefore, rs6413453 mediates TF-binding and may regulate gene expression of *ApoA2*. Previous studies revealed that variation of *ApoA2* gene expression was associated with the size and heterogeneity of HDL particles, while overexpression of human apolipoprotein A-II led to the formation of smaller and heterogeneous HDL particles and formation of small HDL particles was associated with lower HDL cholesterol levels [44–46].

Our study has limitations. First, the number of patients who were homozygous for the minor allele of rs6413453 was relatively small thereby limiting the statistical power of our study. In particular, there has been no GWAS that has identified apolipoprotein A-II as a trait, and the apolipoprotein A-II values used in this study were those at the time of septic shock. Thus, replication of this result in the validation cohort was important in excluding a false positive result. Second, lipoprotein levels were not measured in the Caucasian septic shock cohort so we could not test for replication of association between *ApoA2* genotype and apolipoprotein A-II and HDL levels. Third, in the Mendelian randomization analysis, apolipoprotein A-II concentrations were measured at the time of ICU admission for septic shock and therefore likely were altered from baseline healthy values. This may have made it difficult to identify genetic instruments with a strong correlation to normal apolipoprotein A-II concentrations. Large-scale genetic studies on apolipoprotein A-II are desirable.

## Conclusions

Low apolipoprotein A-II levels are associated with increased mortality in septic shock patients. Genetic variation in *ApoA2* was associated with altered mortality and days free of organ dysfunction in two different ancestries of septic shock patients. Our results implicate decreased apolipoprotein A-II levels as a causal contributor to adverse clinical outcome of septic shock.

## Supplementary Information

Additional file 1.

## Data Availability

The datasets used and analyzed during the current study are available from the corresponding author upon reasonable request and ethics approval.

## Abbreviations

ICU: Intensive care unit
LPS: Lipopolysaccharide
LTA: Lipoteichoic acid
HDL: High-density lipoprotein cholesterol
LDL: Low-density lipoprotein cholesterol
IDL: Intermediate density lipoprotein
VLDL: Very low-density lipoprotein cholesterol
SOFA: Sequential organ failure assessment
SNP: Single nucleotide polymorphisms
VASST: Vasopressin and septic shock trial
UTR: Untranslated region
MAF: Minor-allele frequency
APACHE: Acute Physiology and Chronic Health Evaluation (APACHE)
GWAS: Genome-wide association study
CI: Confidence interval
eQTL: Expression quantitative trait locus
HR: Hazard ratio
