# Supplementary material for: Low apolipoprotein A-II levels causally contribute to increased mortality in septic shock
Source: J Intensive Care. 2025 Feb 20;13:10. doi: 10.1186/s40560-025-00782-2 (PMC11841007; doi:10.1186/s40560-025-00782-2)

Supplementary file

Low Apolipoprotein A-II Levels Causally Contribute to Increased Mortality in Septic Shock.

Authors: Nozomi Takahashi[1,2], Kyle R. Campbell[1], Tadanaga Shimada[2], Taka-aki Nakada[2], James A. Russell[1], Keith R. Walley[1]

Affiliations:

1. Centre for Heart Lung Innovation, St. Paul's Hospital, The University of British Columbia, Vancouver, BC, Canada
2. Chiba University Graduate School of Medicine, Department of Emergency and Critical Care Medicine, Chiba, Japan

Contents:

Methods

Selection of SNPs and genotyping

Cis-eQTL analysis

Table S1. Brussels organ dysfunction definitions

Table S2. Variants from UK Biobank pQTL for baseline apolipoprotein A-II levels

Table S3. *ApoA2* based target gene instruments for eQTL

Table S4. Multivariate logistic regression analysis for the hospital mortality in the derivation cohort

Table S5. Candidate tag SNPs and Hardy-Weinberg equilibrium in *ApoA2*

Table S6. Patients' characteristics in septic shock cohorts by the genotype of *ApoA2* rs6413453 polymorphism

Table S7. Genome-wide association between SNP allele dose and apolipoprotein A-II levels in Japan cohort

Table S8. Differential gene expression for *ApoA2* between septic shock patients and control patients.

Figure S1. Tag SNPs and linkage disequilibrium for two each population in *ApoA2*
Figure S2. Meta-analysis of cox proportional hazard ration combined with Japan and VASST cohort.
Figure S3. Comparison of apolipoprotein A-II levels by each genotype.
Figure S4. Mendelian randomization analyses for the change in 28-day mortality in septic shock using weighted median and MR-Egger method.
Figure S5. Mendelian randomization analyses for the change in 28-day mortality using pQTL traits from UK biobank as instruments.
Figure S6. Inverse variance weighted model for VASST cohort using *ApoA2* cis-eQTL variants.

Methods

Selection of SNPs and genotyping

Haplotype tag SNPs for the candidate gene were identified from the gene region extending from 2,000 bp upstream of the 5' untranslated region (UTR) to 2,000 bp downstream of the 3' UTR since tag SNPs are able to represent differences due to SNPs in the corresponding gene. We considered SNPs with a minor-allele frequency (MAF) > 5% and r^2 threshold was set at 0.8 using TAGster to choose tag SNPs [1]. Tag SNPs for our Japan cohort were chosen from HapMap Phase 2+3 of Japan in Tokyo (JPT) and tag SNPs for our Caucasian cohort were chosen from HapMap Phase 2+3 of Utah residents with Northern and Western European ancestry from the Centre d'Etude du Polymorphisme Humain collection data (International HapMap Project [ftp.ncbi.nlm.nih.gov/hapmap/]). We tested for Hardy-Weinberg equilibrium using a χ^2 test as a data quality check.

DNA was extracted from buffy coat of discarded blood samples using a QIAamp DNA Mini Kit for the Japan cohort and QIAmp DNA Blood Midi kit for the VASST cohort (Qiagen, Hilden, Germany) according to the manufacturer's instructions and genotyped using the Illumina Human Omni or Illumina Infinium Omni in the Japan cohort. All SNPs were genotyped as part of whole genome genotyping using the Illumina Human 1M-Duo genotyping platform in the VASST cohort.

The selected SNPs were confirmed to be functional by Clinvar, PhenoScanner and RegulomeDB which scores SNP's functionality with grades from 1 to 6 based on an alteration of transcription factor binding and a gene regulatory effect [2, 3].

1. Xu Z, Kaplan NL, Taylor JA. TAGster: efficient selection of LD tag SNPs in single or multiple populations. *Bioinformatics*. 2007;23(23):3254-3255. doi:10.1093/bioinformatics/btm426
2. Boyle AP, Hong EL, Hariharan M, et al. Annotation of functional variation in personal genomes using RegulomeDB. *Genome Res*. 2012;22(9):1790-1797. doi:10.1101/gr.137323.112
3. Dong S, Zhao N, Spragins E, et al. Annotating and prioritizing human non-coding variants with RegulomeDB v.2. *Nat Genet*. 2023;55(5):724-726. doi:10.1038/s41588-023-01365-3

Cis-eQTL analysis

To examine the effect of *ApoA2* gene expression on outcome, we extracted cis-eQTL variants for *ApoA2* using the eQTL database [4] and used these as genetic variants to

analyze the association with 28-day mortality using two-sample Mendelian randomization. This database does not provide beta coefficient and standard error, and requires conversion from Z scores using the European population, so only the VASST cohort was used in the analysis. The beta coefficient and standard error were calculated from the Z score using the following formula (*p*, effect allele frequency; *n*, sample size), where the European population in the 1000 genome project were used as the reference panel [5].

$$beta = \frac{z_{score}}{\sqrt{2p(1-p)\left(n + {Z_{score}}^2\right)}}$$

$$SE = \frac{1}{\sqrt{2p(1-p)\left(n + {Z_{score}}^2\right)}}$$

The resulting list of cis-eQTLs was clumped using the same reference panel with a window size of 250 bp and r^2=0.3 (**Table S3**).

4. Võsa U, Claringbould A, Westra HJ, et al. Large-scale cis- and trans-eQTL analyses identify thousands of genetic loci and polygenic scores that regulate blood gene expression. *Nat Genet*. 2021;53(9):1300-1310. doi:10.1038/s41588-021-00913-z

5. Zhu Z, Zhang F, Hu H, et al. Integration of summary data from GWAS and eQTL studies predicts complex trait gene targets. *Nat Genet*. 2016;48(5):481-487. doi:10.1038/ng.3538

Table S1. Brussels organ dysfunction definitions

			Clinically Significant Organ Dysfunction		
Organs	Normal	Mild	Moderate	Severe	Extreme
Cardiovascular (systolic blood pressure, mmHg)	>90	≤ 90<br>Fluid responsive	≤ 90<br>not fluid responsive	≤ 90<br>pH 7.3	≤ 90<br>pH 7.2
Pulmonary (PaO_2/FIO_2, mmHg)	>400	301-400	201-300	101-200	100
Neurologic (GCS)	15	13-14	10-12	6-9	≤ 5
Coagulation (platelet count, $x10^3/mm^3$)	>120	81-120	51-80	21-50	≤20
Renal (creatinine, µmol/L)	<133	133-175	176-300	301-442	≥443
Hepatic (bilirubin, µmol/L)	<20	20-32	33-99	100-199	≥200

Days alive and free (DAF) calculations. DAF was scored as 1 if the patient was alive and free of organ dysfunction (normal or mild dysfunction). DAF was scored as 0 if the patient had organ dysfunction (moderate, severe, or extreme) or was not alive. Each of the 28 days after meeting the inclusion criteria was scored. For any 24-hour period in which there is no measurement of a variable, we carried forward the value from the previous 24-hour period. If a variable was never measured, it was assumed to be normal. Once a patient was discharged home, they were considered free of organ failure.

Table S2. Variants from UK Biobank pQTL for baseline apolipoprotein A-II levels.

Chr	Position	SNP	Gene	EA	OA	EAF	Beta	SE	*P* value
1	161,230,697	rs4073054	MIR5187	C	A	0.63	0.067	0.008	5.59E-19
1	177,969,302	rs6682862	SEC16B	G	A	0.17	-0.076	0.01	7.86E-15
2	20,174,397	rs11096641	RNU6-961P	G	A	0.47	-0.089	0.007	1.44E-34
4	102,267,552	rs13107325	SLC39A8	C	T	0.07	-0.145	0.014	1.57E-25
8	9,326,721	rs4240624	PPP1R3B-DT	G	A	0.91	0.121	0.013	1.36E-21
8	125,487,789	rs28601761	-	C	G	0.42	0.055	0.007	1.28E-13
9	104,888,062	rs62568182	ABCA1	T	G	0.12	-0.086	0.011	1.93E-14
9	136,493,224	rs12555241	NOTCH1	G	A	0.28	-0.059	0.008	8.17E-13
11	116,840,425	rs2727784	SIK3	C	T	0.65	-0.057	0.008	1.32E-13
15	60,591,082	rs339969	RORA-AS1	C	A	0.62	0.052	0.007	2.91E-12
16	56,953,853	rs56228609	-	C	T	0.32	0.095	0.008	1.55E-33
19	19,268,740	rs58542926	TM6SF2	C	T	0.08	0.103	0.014	8.10E-14
19	44,908,684	rs429358	APOE	T	C	0.16	0.127	0.01	2.21E-36

EA, effect allele; OA, other allele; EAF, effect allele frequency; SE, standard error

Table S3. ***ApoA2*** **based target gene instruments for eQTL**

Chr	Position	SNP	EA	OA	*n*	*P* value	Z score	EAF	Beta	SE
1	161178684	rs3924264	C	T	29432	6.71E-54	16.6169	0.392	0.140	0.0084
1	161184097	rs1136224	G	A	29103	2.73E-10	8.7494	0.185	0.093	0.0107
1	161186303	rs12094497	A	G	29317	1.78E-11	9.0526	0.094	0.128	0.0141

EA, effect allele; OA, other allele; n, sample size; EAF, effect allele frequency; SE, standard error

Table S4. Multivariate logistic regression analysis for the hospital mortality in the derivation cohort

Model A. Apolipoprotein A-I

	Odds ratio (95% CI)	*P* value
Age, per year	1.02 (1.00-1.03)	0.021
Male sex	1.00 (0.65-1.53)	0.99
APACHE II score	1.08 (1.05-1.12)	< 0.001
SOFA score	1.07 (1.00-1.15)	0.041
Apolipoprotein A-I	0.99 (0.99-1.00)	0.060

Model B. Apolipoprotein A-II

	Odds ratio (95% CI)	*P* value
Age, per year	1.02 (1.00-1.03)	0.031
Male sex	1.01 (0.66-1.56)	0.96
APACHE II score	1.08 (1.05-1.12)	< 0.001
SOFA score	1.08 (1.01-1.15)	0.032
Apolipoprotein A-II	0.95 (0.92-0.98)	< 0.001

Model C. Apolipoprotein C-III

	Odds ratio (95% CI)	*P* value
Age, per year	1.02 (1.00-1.03)	0.022
Male sex	1.02 (0.66-1.58)	0.92
APACHE II score	1.09 (1.05-1.13)	< 0.001
SOFA score	1.07 (1.00-1.14)	0.057
Apolipoprotein C-III	0.97 (0.92-1.02)	0.20

1) APACHE, acute physiology and chronic health evaluation
2) SOFA, sequential organ failure assessment

Table S5. Candidate tag SNPs and Hardy-Weinberg equilibrium in *ApoA2*

SNP ID	GRCh37 position	Gene region	Major/minor allele	Average MAF[1)]	Average r^2	SNP captured[2)]	HWE *P*-Value[3)] (Japan/VASST)
rs3829793	chr1:161224610	upstream	C/G	0.3903	0.98	CEU, JPT	0.050/0.54
rs5082	chr1:161223893	upstream	G/A	0.388	1	JPT	0.25/0.71
rs5085	chr1:161222721	intronic	C/G	0.2205	1	CEU, JPT	0.38/0.90
rs6413453	chr1:161222526	intronic	G/A	0.228	1	CEU, JPT	0.40/0.98
rs12721035	chr1:161191339	downstream	C/T	0.2073	1	CEU, JPT	0.40/0.82

1) MAF, minor allele frequency
2) JPT, Japanese in Tokyo; CEU, Centre d'Etude du Polymorphisme Humain collection
3) Hardy-Weinberg equilibrium was tested using a χ^2 test with Yate's correction in VASST.

Table S6. Patients' characteristics in septic shock cohorts by the genotype of *ApoA2* rs6413453 polymorphism

	Japan cohort (n = 513)			VASST cohort (n = 474)		
ApoA2 rs6413453 Genotype	GG (n = 320)	AG/AA (n = 193)	P-value	GG (n = 392)	AG/AA (n = 82)	P-value
Age, yr	70 (59-77)	69 (57-75)	0.28	64 (52-74)	68 (53-74)	0.27
Male sex, n (%)	202 (63.1)	139 (72.0)	0.043	156 (39.8)	33 (40.2)	1.00
BMI, kg/m^2	23 (20-26)	22 (20-25)	0.20	28 (24-33)	26 (23-31)	0.073
APACHE II score 1)	29 (23-37)	30 (24-36)	0.73	26 (21-31)	26 (22-32)	0.63
Surgical, n (%)	58 (18.1)	46 (23.8)	0.14	88 (22.4)	17 (20.7)	0.92
Acute low-dose corticosteroids, n (%)	86 (26.9)	63 (32.6)	0.19	206 (52.6)	44 (53.7)	0.90
Variables on day1						
Body temperature, C	37.4 (36.5-38.4)	37.2 (36.5-38.5)	0.55	38.5 (37.6-39.2)	38.6 (37.7-39.1)	0.81
Heart rate, /min	112 (96-129)	109 (94-127)	0.39	125 (110-139)	128 (111-144)	0.64
MAP, mmHg 2)	73 (59-89)	72 (60-91)	0.69	55 (50-61)	57 (49-62)	0.53
WBC, $*10^3/mm^3$ 3)	11.5 (6.1-17.8)	11.5 (5.8-17.3)	0.77	14.1 (8.1-21.1)	13.0 (6.5-21.4)	0.67
Platelet, $*10^3/mm^3$	134 (70-226)	126 (77-225)	0.75	173 (92-265)	174 (96-270)	0.85
Creatinine, µmol/L	127 (75-231)	130 (75-205)	0.87	151 (90-260)	146 (90-240)	0.50
Lactate, mmol/L	3.2 (1.9-5.9)	2.9 (1.7-6.0)	0.23	1.8 (1.0-3.6)	1.6 (0.8-2.8)	0.20

Data are median (interquartile range) for continuous variables.

P values were calculated using Pearson's chi-square test and Mann-Whitney U test.

1) APACHE, acute physiology and chronic health evaluation
2) MAP, mean arterial pressure
3) WBC, white blood cell

Table S7. Genome-wide association between SNP allele dose and apolipoprotein A-II levels in Japan cohort

Chr	Position	SNP	Beta	SE	r^2	T-statistic	*P*-value
2	16498117	rs340786	4.985	0.9272	0.06646	5.376	1.29E-07
16	17846570	rs13330944	2.287	0.4844	0.05193	4.722	3.22E-06
18	68106862	rs28662334	4.357	0.9404	0.05009	4.633	4.86E-06
6	29589666	rs29220	-2.231	0.4868	0.04909	-4.584	6.08E-06
13	72451031	rs76733271	2.346	0.515	0.04907	4.554	6.98E-06
6	23301437	rs1925459	3.457	0.7641	0.048	4.524	7.96E-06
6	130194422	rs11752628	-2.169	0.4816	0.0476	-4.505	8.70E-06
19	53596169	rs6509724	-2.372	0.528	0.04746	-4.492	9.21E-06
4	125907938	rs79796177	5.107	1.139	0.04718	4.484	9.56E-06

Chr, Chromosome; SE, standard error

Table S8. Differential gene expression for *ApoA2* between septic shock patients and control patients.

Probe ID	t-statistic	B-statistic	log2 fold change	Adjusted *P* value
219465_at	12.1972976	42.038966	1.27537242	1.75E-21
219466_s_at	6.0374817	8.7369	0.65880691	9.61E-08

Figure S1. Tag SNPs and linkage disequilibrium for two each population in *ApoA2*

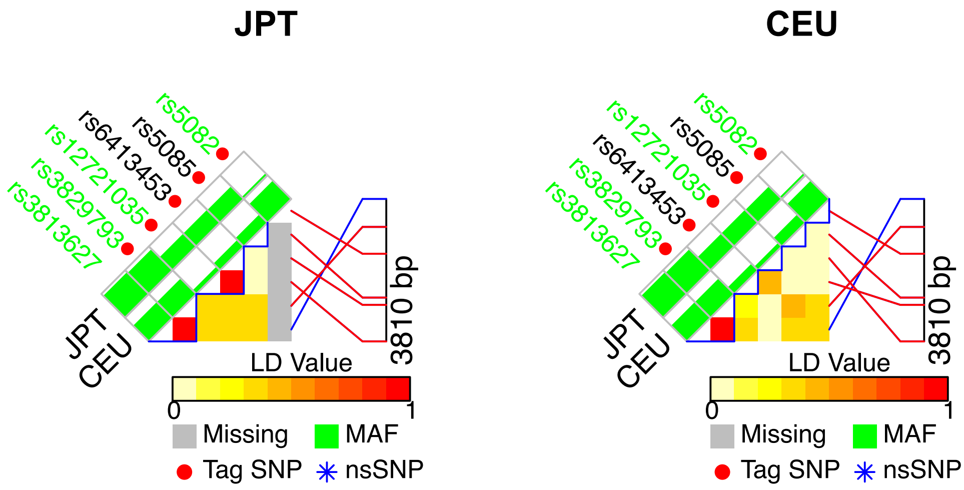

JPT, Japanese in Tokyo; CEU, Centre d'Etude du Polymorphisme Humain collection

Figure S2. Meta-analysis of cox proportional hazard ration combined with Japan and VASST cohort. Random effects model shows significantly higher hazard ratio (HR, 1.71; 95% CI, 1.20–2.44; *P* = 0.0031).

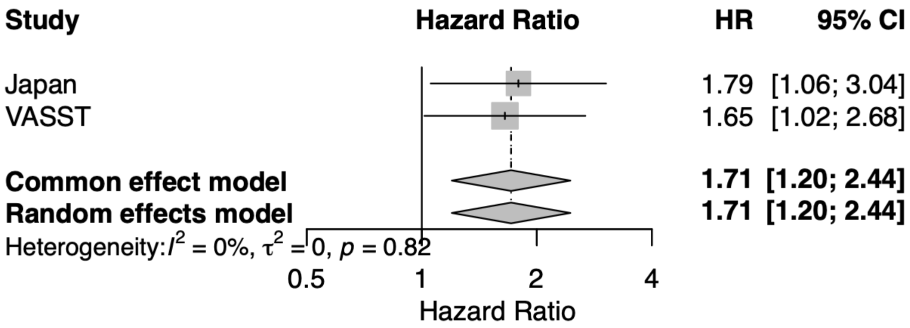

Figure S3. Comparison of apolipoprotein A-II levels by each genotype. There was no significant difference between AG/AA (12.4 [IQR 8.7, 18.7] mg/dL) and GG (11.3 [7.7, 15.8] mg/dL) (*P*=0.069).

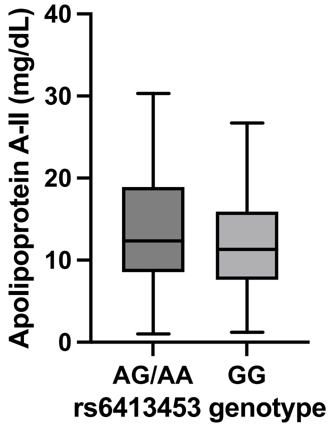

Figure S4. Mendelian randomization analyses for the change in 28-day mortality in septic shock using weighted median and MR-Egger method.

A. **Weighted median method. The odds ratio of 28-day mortality to a decrease in apolipoprotein A-II 1 mg/dL was 1.02 (95% CI; 1.01-1.03, *P*=0.025).** X-axis: changes in apolipoprotein A-II (mg/mL) per allele change; Y-axis: changes in natural logarithm (ln) odds per allele change.

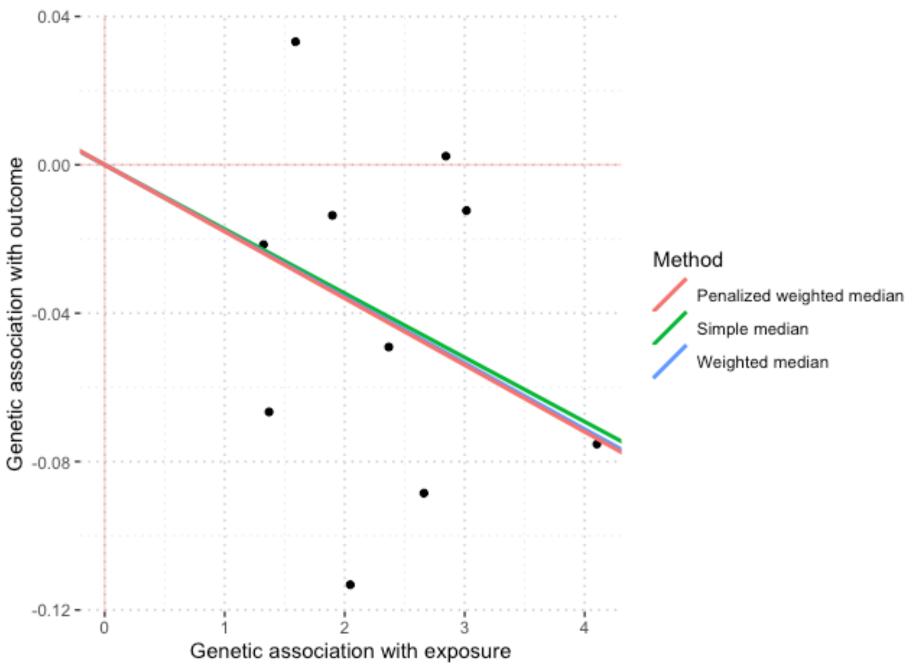

B. **MR-Egger method. The estimate of the intercept was 0.036 (95% CI, -0.065–0.136; *P* = 0.49).** X-axis: changes in apolipoprotein A-II (mg/mL) per allele change; Y-axis: changes in natural logarithm (ln) odds per allele change. Two of the four lines are not visible due to overlap.

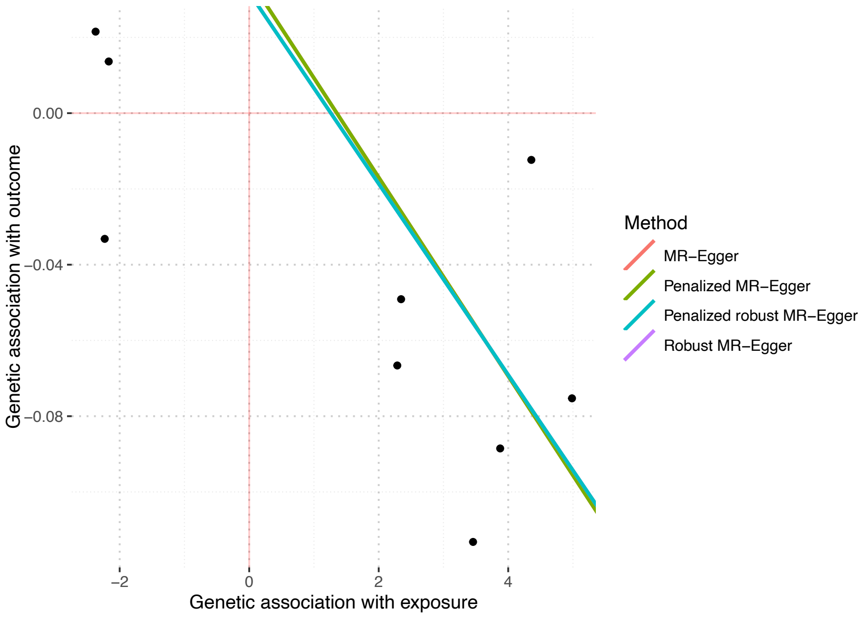

Figure S5. Mendelian randomization analyses for the change in 28-day mortality using pQTL traits from UK biobank as instruments.

(A) Inverse variance weighted model for Japan cohort. The odds ratio of 28-day mortality to a decrease in apolipoprotein A-II 1 mg/dL was 1.54 (95% CI; 0.13–18.2, *P*=0.72).

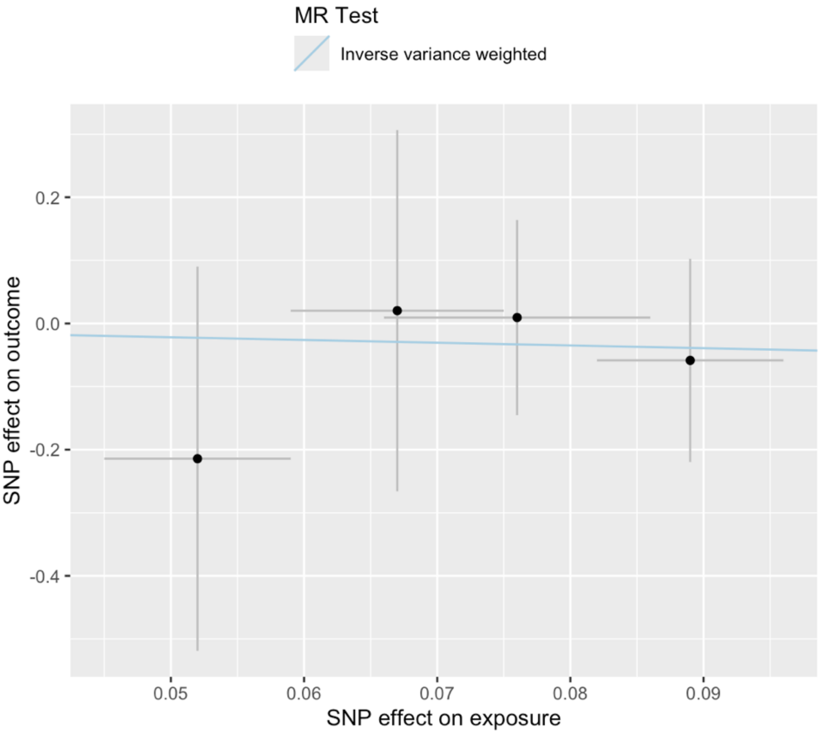

(B) Inverse variance weighted model for VASST cohort. The odds ratio of 28-day mortality to a decrease in apolipoprotein A-II 1 mg/dL was 1.79 (95% CI; 0.34–9.42, *P*=0.49).

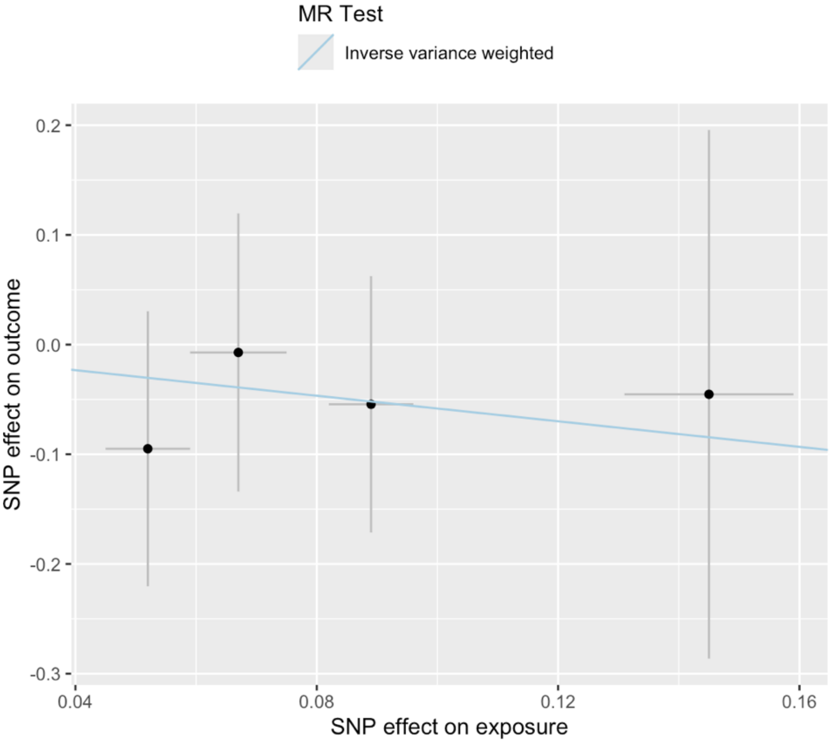

Figure S6. Inverse variance weighted model for VASST cohort using *ApoA2* cis-eQTL variants.

The odds ratio of 28-day mortality to a decrease in *ApoA2* was 4.88 (IVW, 95% CI; 1.40–17.01, *P*=0.013), 3.94 (weighted median, 95% CI; 1.06–14.72, *P*=0.034), 4.64 (MR-Egger, 95% CI; 2.02×10^{-3}–1.18×10^{4}, *P*=0.70 and 3.81 (weighted mode 95% CI; 0.94–15.52, *P*=0.79).

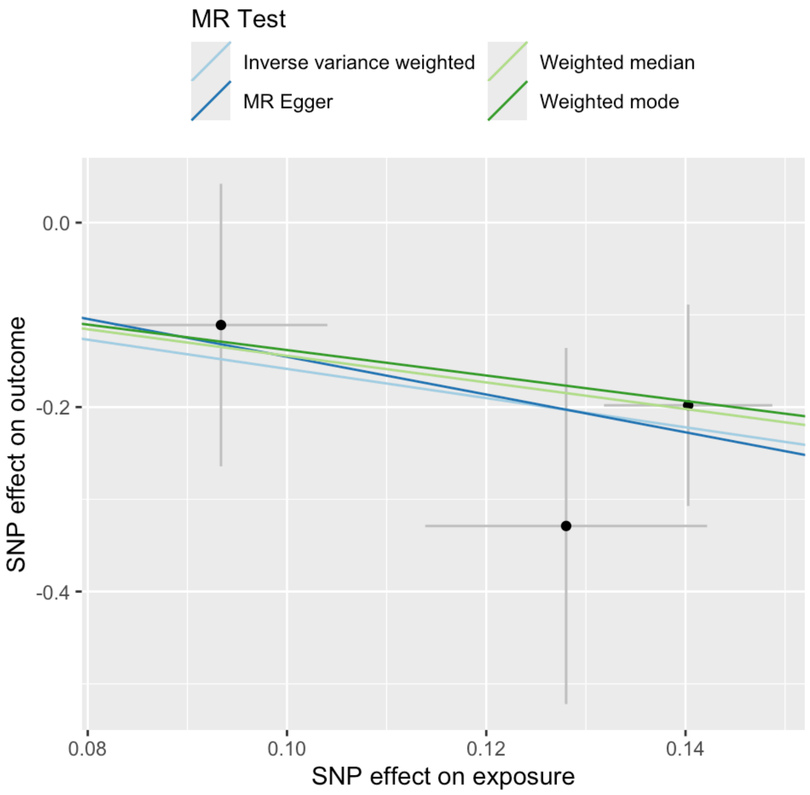

Supplement: Supplementary file 1 — Additional file 1. [file 40560_2025_782_MOESM1_ESM.pdf]
